# From petals to healing: consolidated network pharmacology and molecular docking investigations of the mechanisms underpinning *Rhododendron arboreum* flower’s anti-NAFLD effects

**DOI:** 10.3389/fphar.2024.1366279

**Published:** 2024-05-28

**Authors:** Nitish Singh Jangwan, Mausin Khan, Richa Das, Najla Altwaijry, Ahlam Mansour Sultan, Ruqaiyah Khan, Shakir Saleem, Mamta F. Singh

**Affiliations:** ^1^ Department of Pharmacognosy and Phytochemistry, School of Pharmaceutical Sciences, Delhi Pharmaceutical Sciences and Research University, New Delhi, India; ^2^ Department of Pharmaceutical Chemistry, School of Pharmaceutical Sciences and Technology, Sardar Bhagwan Singh University, Dehradun, Uttarakhand, India; ^3^ Department of Biotechnology, Parul Institute of Applied Science, Parul University, Vadodara, Gujarat, India; ^4^ Department of Pharmaceutical Sciences, College of Pharmacy, Princess Nourah Bint Abdulrahman University, Riyadh, Saudi Arabia; ^5^ Department of Basic Health Sciences, Deanship of Preparatory Year for the Health Colleges, Princess Nourah Bint Abdulrahman University, Riyadh, Saudi Arabia; ^6^ Department of Public Health, College of Health Sciences, Saudi Electronic University, Riyadh, Saudi Arabia; ^7^ College of Pharmacy, COER University, Roorkee, Uttarakhand, India

**Keywords:** burans, cytoscape, hepatoprotective, quercetin, rutin

## Abstract

Rhododendron arboreum: Sm., also known as Burans is traditionally used as an anti-inflammatory, anti-diabetic, hepatoprotective, adaptogenic, and anti-oxidative agent. It has been used since ancient times in Indian traditional medicine for various liver disorders. However, the exact mechanism behind its activity against NAFLD is not known. The aim of the present study is to investigate the molecular mechanism of *Rhododendron arboreum* flower (RAF) in the treatment of NAFLD using network pharmacology and molecular docking methods. Bioactives were also predicted for their drug-likeness score, probable side effects and ADMET profile. Protein-protein interaction (PPI) data was obtained using the STRING platform. For the visualisation of GO analysis, a bioinformatics server was employed. Through molecular docking, the binding affinity between potential targets and active compounds were assessed. A total of five active compounds of RAF and 30 target proteins were selected. The targets with higher degrees were identified through the PPI network. GO analysis indicated that the NAFLD treatment with RAF primarily entails a response to the fatty acid biosynthetic process, lipid metabolic process, regulation of cell death, regulation of stress response, and cellular response to a chemical stimulus. Molecular docking and molecular dynamic simulation exhibited that rutin has best binding affinity among active compounds and selected targets as indicated by the binding energy, RMSD, and RMSF data. The findings comprehensively elucidated toxicity data, potential targets of bioactives and molecular mechanisms of RAF against NAFLD, providing a promising novel strategy for future research on NAFLD treatment.

## Graphical Abstract

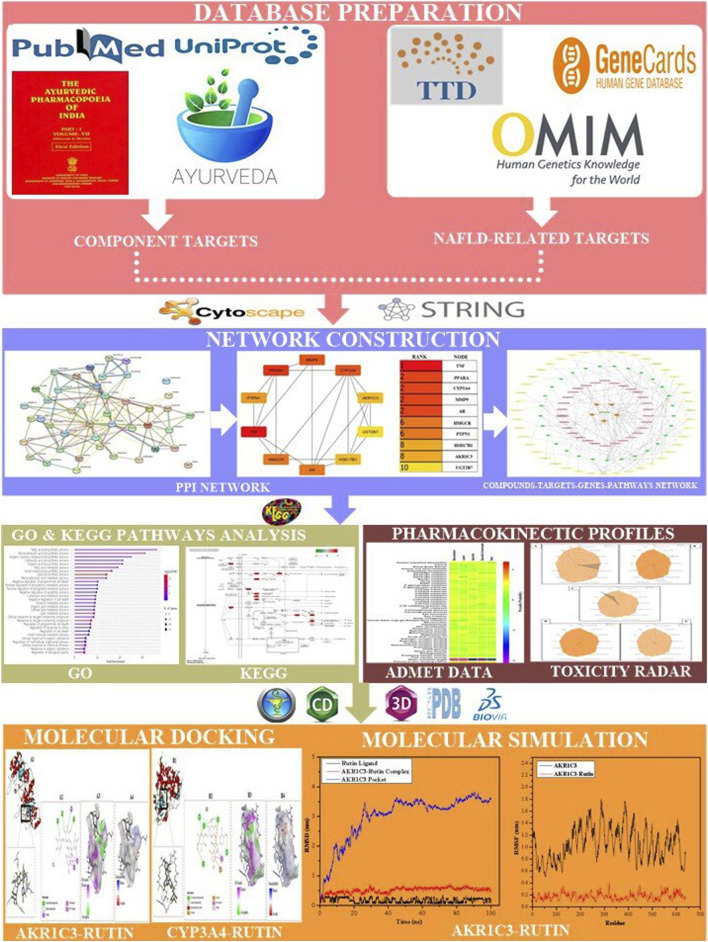

## 1 Introduction

Nonalcoholic fatty liver disease (NAFLD) is an array of ailments marked by obese liver intrusion, steatosis, steatohepatitis, and cirrhosis (Remya, 2018; Sahu et al., 2022). NAFLD may advance from basic steatosis to steatohepatitis and fibrosis. Its fatal consequences may be cirrhosis or hepatocellular carcinoma (Singhal et al., 2015). Considering prevalence rates in the general population spanning from 11.2% to 37.2%, the disease is on the rise globally as a result of an increase in obesity (Benedict and Zhang, 2017). Although there are currently no specific agents designated for the treatment of NAFLD and its progressed forms, a number of intriguing agents such as glucose-lowering drugs, antioxidants, statins and others have been intensively studied over the past several decades (Mantovani and Dalbeni, 2021). Primary treatment approaches involve empirical strategies such as dietary restriction, physical activity, and weight loss (Nseir et al., 2014; Romero-Gómez et al., 2017). Thus, there is a hunt for safe and economically viable alternative forms of therapy in various disciplines of medicine (Remya, 2018). India is dubbed as a global medicinal garden due to its abundant biodiversity and substantial collection of herbal remedies (Verma et al., 2020). NAFLD is one of the non-communicable diseases for which Ayurveda has enormous potential in its therapy (Remya, 2018). Within the framework of Ayurveda, NAFLD is characterized as *Yakrit Roga*, a liver ailment that exhibits promise of remedy through the utilization of herbal medications (Sahu et al., 2022).

*Rhododendron arboreum* Sm., commonly referred to as Burans, is a member of the Ericaceae family. This botanical species holds prestigious distinctions, serving as the national flower of Nepal, the state tree of Uttarakhand (India), and the state flower of Nagaland (India). The processed juice of its flowers, known as rhodojuice or sharbat, has acquired widespread market popularity. Traditionally the flowers are used in the treatment of diarrhoea, dysentery, and dyspepsia, and are highly useful for diseases like diabetes and chronic heart diseases (Ahmad et al., 2022). Flowers, bark, and foliage of *R. arboreum (Rhododendron arboreum)* are utilised to derive multiple phytochemicals. The different pharmacological activities of *R. arboreum* flower, such as anti-oxidant, anti-diabetic, adaptogenic, antiviral, antifungal, antitumor, anti-hyperlipidemic, and anti-inflammatory effects are due to the presence of quercetin, quercetin-3-rhamnoside, rutin, coumaric acid, phenolic compounds, and amino acids (Qiang et al., 2011; Srivastava, 2012; Verma et al., 2020). Rutin shows anti-inflammatory properties by inhibiting the release of phospholipase A2 (PLA2), LOX (lipoxygenase), neutrophil glucuronidase, TNF-α (Tumour Necrosis Factor Alpha), IL-6 (Interleukin 6), and IL-1β (Interleukin-1 beta). Further, it also inhibits the activation of ERK (Extracellular signal-regulated kinase) and p38 and the expression of iNOS (Inducible nitric oxide synthase) in lipopolysaccharide (LPS) stimulated cells (García-Lafuente et al., 2009). *Rhododendron arboreum* methanolic extracts demonstrated anti-inflammatory and anti-nociceptive properties in arachidonic acid-induced hind paw edema, Freund’s adjuvant-induced paw arthritis and cotton pellet granuloma model of inflammation (Verma et al., 2010). *Rhododendron arboreum* is a well-known plant for its hepatoprotective effect (Verma et al., 2011). A research study in rodents indicates the *in vivo* hepatoprotective activity of the ethanolic extract of *R. arboreum* leaves which protects the rodents from carbon tetrachloride (CCl_4_)-induced hepatic injury along with reducing triglyceride and liver cholesterol levels (Prakash et al., 2008). The ethyl acetate portion of *R. arboreum* reduced the increased levels of SGOT (glutamic oxaloacetic transaminase), SGPT (glutamate pyruvate transaminase), GST (glutathione S-transferase), γ-GT (γ-glutamyl transferase), etc., to indicate hepatoprotective effect against (CCl_4_)-induced hepatic injury (Verma et al., 2011). No research work to date has probed the role of *R. arboreum* in NAFLD management. The current research work makes an effort to ascertain the possible phytoconstituents and molecular targets related to NAFLD in the *R. arboreum* flower (RAF). Employing network pharmacology, it highlights how the possible phytoconstituents interact with the emergent protein targets and signaling pathways related to NAFLD. The mode of binding of the phytoconstituents and the protein targets in molecular docking, along with the binding affinity, indicates the possible active conformation of the phytoconstituents.

## 2 Materials and methods

### 2.1 Mining of phytoconstituents and proteins involved in NAFLD

The RAF’s phytoconstituents were investigated from the freely accessible literature eBook: “Himalayan Medicinal Plants Advances in Botany, Production and Research,” “Folk-medicine and Aromatic Plants of Uttaranchal,” and several additional references, scientific periodicals mostly open access databases, and Ayurveda eBooks. The search terms included Himalayan medicinal plants, *R. arboreum* known as Burans, Laligurans, Gurans, folk medicine, ethnopharmacology of Burans, and Yakrit Roga to explore information. The phytoconstituents, their Simplified Molecular-Input Line-Entry System (SMILES) categories, and PubChem Compound Identification (PubChem CID) were identified and used to develop a database for screening. Throughout the development of the database, duplicate phytochemicals were eliminated. Each phytoconstituent’s canonical SMILES and PubChem CID were obtained from the PubChem Database (Kim et al., 2016). Using the BindingDB database webtool (open source license), protein targets of the desired ligands were identified by hitting the “Find My Compound’s Target” button under the “Special tools” section on the left. The biological targets in BindingDB were identified by their SMILES, which included a 70% similarity filter, considering existing ligand molecules. Upon entering 0.7 into the similarity column under the “Find My Compound’s Target” tab, the filter was activated (Gilson et al., 2016). There are things that are taken into account by this filter, like how well this existing ligand matches the target protein’s importance, which gives a good balance between specificity, sensitivity, similarity, and diversity. This threshold of a 70 percent similarity filter is sufficient and preserves the structural framework necessary for binding sites, as evidenced by prior work (Liu et al., 2007; Chatterjee et al., 2023). Further, proteins involved in NAFLD were discovered through the use of the Therapeutic Target Database (TTD) web tool with an open source license (Li et al., 2018). By selecting the option “UniportKB/SwissProt”, the “Primary ID” recognized the target protein by navigating to the link underneath the “Hits (All Compounds)" segment. The acquired “Primary ID” grants access to the UniProt database for the selected target protein. Essentially, the UniProt database offers gene IDs for each protein present in the molecule responsible for NAFLD (Apweiler, 2008). With the combined data from BindingDB and TTD, a network can be created that relates a variety of things, including therapeutic linkages and the interaction between phytoconstituents and proteins. These two databases were compatible with each other primarily because, when applicable, there were common identifiers or attribute names that described similar things, for instance, the term “target proteins”. Therefore, the linkages were created on the basis of standardized identifiers: UniProt ID, UniProt Gene, for proteins and canonical SMILES, and PubChem CID for the phytoconstituents.

### 2.2 Drug-likeness prediction and ADMET characteristics

Phytoconstituents’ drug-likeness score was calculated using the MolSoft webtool (http://www.molsoft.com/) open source license. Moreover, the ADMET profiles for each phytoconstituent were predicted using the admetSAR2.0 webtool open source license. Additionally, to determine the ADMET profiles for each phytoconstituent, the SMILES of each phytoconstituent were entered in the “Draw molecule” tab. Next, on the right of the admetSAR2.0 webtool interface, the “Predict” tab was chosen (Yang et al., 2017). In order to check the model’s compound validity, the authors examined its training sets for certain characteristics. All compounds were considered acceptable if their molecular weight and alogP were within the 99% range. Anything that fell outside of this range or went beyond the limits of training set was flagged. For properties like atoms, rings, H-bond acceptors, and donors, only values higher than defined thresholds were flagged. This approach makes it easier to spot molecules whose physicochemical characteristics deviate significantly from the expected ranges in the training dataset. This classification serves as a warning, suggesting that the model’s predictions may not be as accurate or that they may not be applicable to the domain of use that was previously mentioned. Under the heading “In domain,” the work highlights findings that provide significant credibility and validation to the data provided. The bioavailability (F30%) and solubility of the phytoconstituents were predicted using the SwissADME web tool (licensee owner: Molecular Modeling Group of the Swiss Institute of Bioinformatics) (http://www.swissadme.ch). Data on bioavailability and solubility were obtained by entering the SMILES of each phytoconstituent into the “Enter a list of SMILES here” section on the right-hand side of the page and then clicking the “Run” tab below (Daina et al., 2017).

### 2.3 Prediction of side effects

By searching the SMILES notation of each phytoconstituent, the ADVERpred web tool (open source license) was utilised to predict probable side effects (Ivanov et al., 2018). The elimination of molecular charges from phytoconstituents, when relevant, was carried out as part of the process of anticipating any adverse implications. A general goal of de-ionising phytoconstituents is to standardise input data, streamline computational analyses, and possibly improve the precision and dependability of structural feature-based toxicity predictions over charge-related ones (Khanal et al., 2019; Filimonov et al., 2020; Agyapong et al., 2021). The potential for adverse effects was meticulously considered wherever the probability of action (Pa) exceeded the likelihood of inactivity (Pi) and the Pa value was greater than the cutoff of 0.70. ProTox-II, which is a free webtool (https://tox-new.charite.de/protox_II) was used to learn about the toxicity class, toxic doses (LD50 value), and partition coefficient (Log P) (Banerjee et al., 2018). By accessing the “TOX PREDICTION” tab in ProTox-II, entering the target SMILES under the “Canonical Smiles” tab, and selecting the toxicity models of interest, the toxicity of the desired phytoconstituent was determined. To predict the toxicity of the specified phytoconstituent, click “Start Tox-Prediction” at the bottom of the page. Although ProTox-II provides information on chemical substance toxicity and ADMET characteristics, keep in mind that these forecasts are based on computer models that act as a preliminary screening tool. To confirm these predictions before making final opinions in drug development or chemical risk evaluation, experimental validation and additional investigations are crucial.

### 2.4 Pathway and network analysis

In order to identify which pathways were affected by the phytoconstituents, the STRING (Search Tool for the Retrieval of Interacting Genes and Proteins) database (Version: 12.0) was searched for a set of NAFLD-related proteins along with the gene enrichment analysis. The genes linked to the target proteins were entered into the “List of Names” section of the STRING database. From the “Organisms” section, *Homo sapiens* was chosen to represent the protein-protein interaction (PPI) among the genes of NAFLD that were targeted by RAF phytoconstituents (Szklarczyk et al., 2021). To create a thorough network that connected phytoconstituents, protein molecules, and the identified pathways of interest, Cytoscape v3.10.0 was utilised (Shannon et al., 2003).

### 2.5 Docking studies

The three-dimensional structure of phytoconstituents was retrieved from the PubChem database (https://pubchem.ncbi.nlm.nih.gov/), optimized, and saved using Chem3D. The target molecules for NAFLD were retrieved from the RCSB (Research Collaboratory for Structural Bioinformatics) (https://www.rcsb.org/) database (Rose et al., 2017). Discovery Studio Visualizer (v21.1.0.20298) was employed to facilitate the removal of water molecules and heteroatoms from the protein structure (Dassault Systèmes, 2016). PyRx–Python Prescription 0.8 software (open source software) was used to predict the binding affinity of phytoconstituents with NAFLD target receptors (Dallakyan and Olson, 2015). The identification of active pockets within NAFLD target receptors was accomplished through blind docking methodology. Default settings were used for all the calculations. The ligand-protein interaction was visualized by the discovery studio, which selected the pose with the lowest binding energy *via* docking. The docking active center was prepared with the help of the grid box function within PyRx. The authenticity of the results generated was based on assessing the root-mean-square deviation (RMSD≤2.5 Å) between the docked ligand and the original molecule (Qin et al., 2021). PyRx assists in performing user-friendly virtual screening by helping users with everything from data preparation to job submission and result analysis. The user-friendly interface of the PyRx docking wizard oversimplifies the complex drug discovery process. The essential chemical spreadsheet-like features and robust visualization engine of PyRx make it a valuable tool for structure-based drug design. PyRx is favored due to its superior docking accuracy as compared to other freely available docking software and webtools (MVD: 87%, Glide: 82%, Surflex: 75%, FlexX: 58%) (Kumar et al., 2017).

Molecular libraries prepared from the PubChem database contain 2D structures generated from SMILES. The OpenBabel tool in PyRx provides files in Structured Data Format (SDF) for docking. PyRx visualizes results using a TVTK scene, which portrays molecular structures as ball and stick model. Further, energy minimization is performed for specific or all molecules with the help of OpenBabel which has a graphical user interface (GUI) enabling users to alter energy minimization parameters.

PyRx facilitates the conversion of specific or all molecules into PDBQT format. The small fragments can be removed with the help of OpenBabel.StripSalts and partial charges can be selected from OpenBabel or PyBabel provided by MGLTools. PyRx employs AutoDock software for performing molecular docking procedures (Dallakyan and Olson, 2015; Qazi et al., 2021).

### 2.6 Molecular dynamics simulation

The binding score between protein targets and active compounds resulting from molecular docking (docking score, 2D and 3D plot of target protein-active compound interaction (Figure 7)) was assessed by performing a 100 ns atomistic molecular dynamics (MD) simulation with the help of the SiBioLead online molecular dynamics simulation platform (https://sibiolead.com/) (a GPU-based high-performance cluster system running on GROningen MAchine for Chemical Simulations (GROMACS) software suite). All the default settings recommended by the platform were used to perform simulations (Sharma et al., 2021). The GROMACS (Version 2020.4) was utilized to perform MD simulation (Abraham et al., 2015). The Pre-Processing Parameters encompass specific settings such as Forcefield (OPLS/AA), Water model (SPC), Box Type (Triclinic), Neutralization method using NaCl, and a concentration set at 0.15 M or 150 mM. Energy Minimization Parameters involve utilizing the Steepest Descents algorithm for energy minimization with a total of 5,000 steps. Equilibration Parameters entail two types, NVT/NPT equilibration, with a temperature set to 300 K, pressure at 1 bar, and an equilibration time of 100 ps. The Simulation Parameters include the Leap frog integrator for simulation spanning 100 ns, saving a total of 5,000 frames throughout the simulation duration. The conformational stability and feasibility of root mean square deviation (RMSD) were analyzed in a 100 ns simulation. Also, the residues of the protein play a vital role in achieving a stable conformation for a protein-ligand complex, which can be gauged by using the root mean square fluctuation (RMSF) as a parameter. RMSF measures the average displacement of specific atoms or groups concerning a reference structure. Higher deviations from initial coordinates often indicate simulation non-equilibration. Equilibrated simulations involve stable fluctuation around an average conformation, making it reasonable to compute structure subsets’ fluctuations relative to the simulation’s average structure *via* RMSF (Martínez, 2015). The RMSF plot shows the residues that changed significantly during the MD simulation. More oscillations of the residues are indicated by the peaks in the plot. High values in RMSF represent the protein’s domain’s flexibility (Ghahremanian et al., 2022). The potential energy in the MD simulation reflects the body’s entire intermolecular interaction energy. It also shows the body’s stability and how interactions occur. The system is more stable when the potential energy is lower (Levitt et al., 1995). The radius of gyration (Rg) describes the size of a molecule, or its compactness. Rg measures the average distance from the particle’s center of mass, which gives information on the molecular size and shape’s variation during the simulation. If Rg is large, the structure is likely to be lengthy, but if Rg is small, the form is compact. In general, the smaller Rg in MD simulation implies a more favorable interaction scenario, which is translated into the establishment of a stable and well-packed structure with stronger interactions among the components (Rampogu et al., 2022). The proteins were configured using the ‘Optimized Potentials for Liquid Simulations’ (OPLS) all-atom (AA) force field. Similarly, the simple point charge water model (SPC) was used and assigned atomic partial charges to calculate the electrostatic potentials. The addition of the active components was done after structuring the protein, and the charge neutralization was obtained automatically by integrating hydrogen atoms and counterions. There are four main steps to the MD simulation process: energy minimization, gradual heating, equilibrium, and extended production dynamics. At the outset, proteins and small molecules had their heavy atoms constrained, and 5,000 steps were devoted to optimizing water molecules in an effort to minimize energy. Subsequently, the system underwent a controlled temperature increase from 0 to 300 K and constant pressure of 1 bar over a period of 100 picoseconds. In all the MD simulations, the time step was set to 2 fs. Following the heating phase, the system was allowed to equilibrate for 100 picoseconds under the NVT/NPT ensemble conditions. Employing both consecutive NVT (canonical ensemble) and NPT (isothermal-isobaric ensemble) equilibration steps was crucial for stabilizing the system effectively. This sequential equilibration ensured attainment of a balanced state in temperature and pressure, crucial for the subsequent production of MD runs. Finally, a 100 nanosecond molecular dynamics simulation was conducted, comprising a total of 5,000 steps, also under the NVT/NPT ensemble. A longer simulation time, like the 100 ns duration was utilized in current MD simulations, is preferred for multiple reasons: it broadens the sampling of molecular motion, allowing exploration of slower or infrequent processes; it enhances the likelihood of observing conformational changes, aiding in characterizing diverse states or intermediate structures. Extended durations also facilitate reaching a stable state, ensuring consistent system properties for more precise behavioral insights. In essence, longer simulation times offer a thorough examination of molecular behavior, but their selection must consider computational constraints and align with the study’s scientific objectives (Frenkel et al., 1996).

## 3 Results

### 3.1 NAFLD-related phytoconstituents and proteins

Phytoconstituents of RAF were mined from the available scientific literature. A total of 25 phytoconstituents (nine anthocyanins, six phenolic acids, and ten flavonoids) were identified, of which only lupeol, quercetin, quercetin-3-rhamnoside, rutin, and β–sitosterol were predicted to modulate the NAFLD protein molecules (Table 1). The phytoconstituents (lupeol, quercetin, quercetin-3-rhamnoside, rutin, and β-sitosterol) were assessed for their potential to influence NAFLD-related protein molecules by analyzing them in BindingDB at a 70% similarity threshold. Numerous target proteins were identified to be influenced by these phytoconstituents, but only those specifically associated with the pathogenesis of NAFLD were considered, while others were excluded from the analysis. These bioactives structurally belonged to the flavonoids, terpenoids, or steroids category. Target anticipation in BindingDB (70 targets) was performed employing SMILES at a 70% similarity threshold with recognized ligand compounds. The proteins associated with NAFLD were identified by referencing known targets from the TTD (50 target proteins). After analysis with Binding DB (Binding Database), it became apparent that the bioactives interacted with hundreds of targets implicated in a variety of disorders. Further screening the targets for NAFLD, the RAF bioactives were found to interact with around 30 targets (Supplementary Material).

**TABLE 1:** Types of compounds and their targets.

S.No.	Compound	Compound type	PubChem CID	Targeted proteins
1	Lupeol	Pentacyclic triterpenoid	259846	HSD17B1, AR, NR1H4, NR1H3, UGT2B7, FxR
2	Quercetin	Flavonols	5280343	HSD17B1, HSD17B2, ADORA3, AKR1B1, AKR1C3, AR, NTRK2, MET, IGF1R, GAA, MMP9, PPARA, ALOX12, LOX15, PIK3CG, NOX4, ChREBP, NFκB
3	Quercetin-3-rhamnoside	Flavonols	5280459	ADRA2A, GUSB, CYP3A4, IL2, MMP9, NOX4, ALOX5, TNF, PTPN1, SREBPc, NFκB
4	Rutin	Flavonoid glycoside	5280805	ADORA3, AKR1B1, ADRA2A, GUSB, CYP3A4, IL2, MMP9, NOX4, ALOX5, PTPN1, TNF, ChREBP, FxR, NFκB
5	β -sitosterol	Stigmastane sterol	222284	HSD17B1, HMGCR, PRKAB1, AR, NR1H4, GAA, NR1H3, NR1H2, FxR, SREBPc, NFκB

### 3.2 Predictive side effects, ADMET profile, and drug-likeness of compounds

**FIGURE 1:**
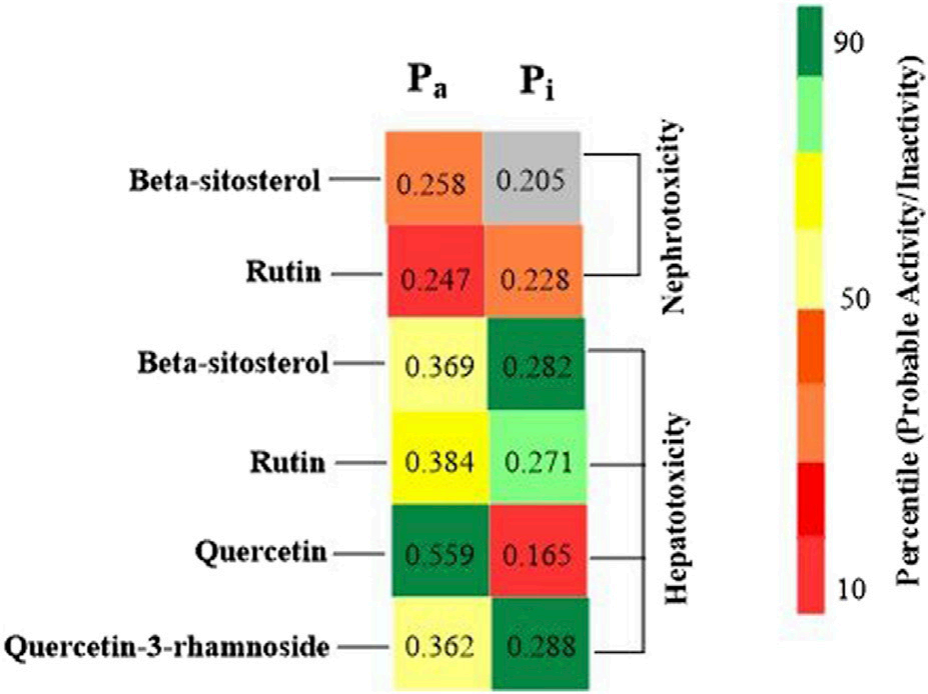
Probable side effects of phytoconstituents (By ADVERpred web tool) P_a_: Probable activity, P_i_: Probable inactivity. The side effects were considered if the probable activity (P_a_) is higher than probable inactivity (P_i_).

Foreseeable side effects to all four phytoconstituents (excluding lupeol) are depicted in Figure 1. Nephrotoxicity and hepatotoxicity risk profiles for anticipated substances are depicted in Figure 1. Predictions were made for the absorption rate of phytochemicals, their capacity to cross the blood-brain barrier, their mutagenicity, their toxicity to fish in the water and their affinity for binding plasma proteins. The Bioavailability (F30%), partition coefficient (Log P) and solubility (Log *S*) of the phytoconstituents were predicted by the SwissADME web tool (Table 2). Additionally, the toxicity (LD_50_) and toxicity class as per the globally harmonized system (GHS) of classification of labelling of chemicals of five phytoconstituents (Table 3) were predicted by ProTox-II web tool. Validation of computational findings was achieved through the correlation of reported experimental data on the phytoconstituents with computational outcomes (e.g., toxicity dose (LD_50_ values) and toxicity class result). The toxicity data of the lupeol phytoconstituent, as reported experimentally (https://pubchem.ncbi.nlm.nih.gov/compound/259846#section=Disposal-Methods; https://echa.europa.eu/information-on-chemicals/cl-inventory-database/-/discli/details/113575HYPERLINK "https://echa.europa.eu/information-on-chemicals/cl-inventory-database/-/discli/details/113575" \o "https://echa.europa.eu/information-on-chemicals/cl-inventory-database/-/discli/details/113575"https://echa.europa.eu/information-on-chemicals/cl-inventory-database/-/discli/details/113575), align with the predicted LD_50_ (2000 mg/kg) and toxicity class (Class 4 according to GHS classification). Similarly, the predicted LD_50_ of quercetin (159 mg/kg), rutin (5,000 mg/kg), and β -sitosterol (890 mg/kg) phytoconstituents is in accordance with the reported toxicity data of quercetin (Sullivan et al., 1951; http://www.t3db.ca/toxins/T3D4758), rutin [(Lambev et al., 1980; https://echa.europa.eu/information-on-chemicals/cl-inventory-database/-/discli/details/54624) and β -sitosterol (Paniagua-Pérez et al., 2005)].

**TABLE 2:** Active compounds, structures and their properties.

S.No.	Phytoconstituents	Structure	Bioavailability (F30%)	Log P	Solubility (Log *S*)	Solubility class
1	Lupeol	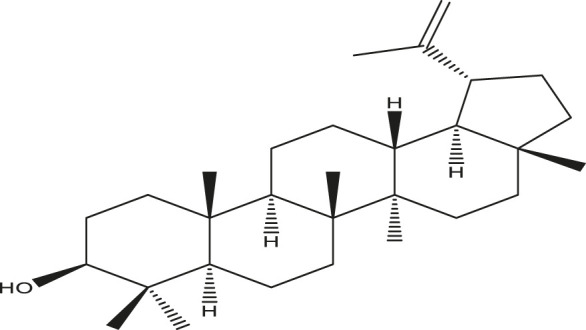	0.55	8.02	−6.74	Poorly soluble
2	Quercetin	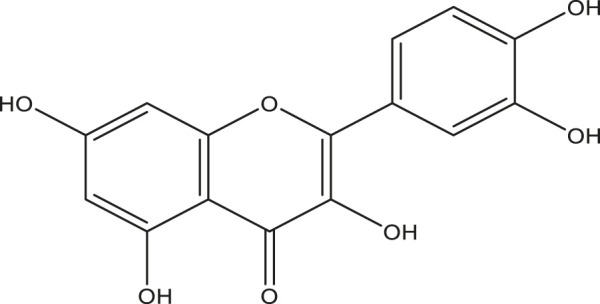	0.55	1.99	−3.24	Soluble
3	Quercetin-3-rhamnoside	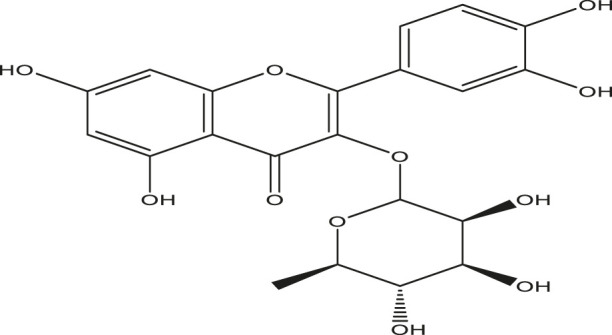	0.17	0.49	−2.08	Soluble
4	Rutin	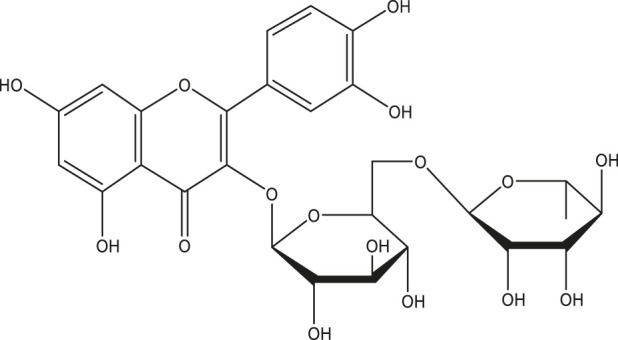	0.17	−1.69	−0.29	Soluble
5	β -sitosterol	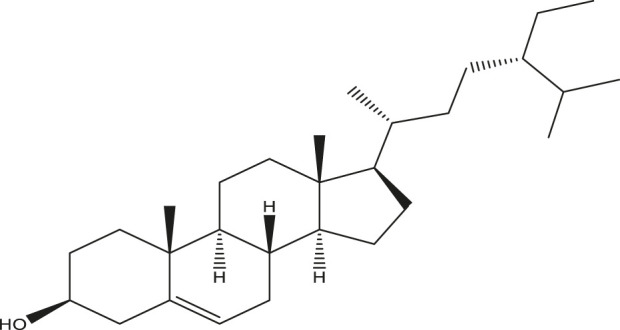	0.55	8.02	−6.19	Poorly soluble

Solubility class: Log *S* scale.

Insoluble < −10 < Poorly soluble < −6 < Moderately soluble < −4 < Soluble < −2 Very soluble <0 Highly soluble.

Molecular structure is drawn by ChemDraw Professional 15.1 software. The Bioavailability (F30%), partition coefficient (Log P) and solubility (Log S) of the phytoconstituents were predicted by the SwissADME, web tool.

**TABLE 3:** Active compounds and their toxicity data (Data generated by ProTox-II web tool).

S.No.	Phytoconstituents	Toxic dose (mg/kg) (Predicted LD_50_)	Toxicity class
1	Lupeol	2000	4
2	Quercetin	159	3
3	Quercetin-3-rhamnoside	5,000	5
4	Rutin	5,000	5
5	β -sitosterol	890	4

Toxicity classes are defined according to the globally harmonized system (GHS) of classification of labelling of chemicals. LD_50_ values are given in [mg/kg].

Class I: fatal if swallowed (LD_50_ ≤ 5).

Class II: fatal if swallowed (5 < LD_50_ ≤ 50).

Class III: toxic if swallowed (50 < LD_50_ ≤ 300).

Class IV: harmful if swallowed (300 < LD_50_ ≤ 2000).

Class V: may be harmful if swallowed (2000 < LD_50_ ≤ 5,000).

Class VI: non-toxic (LD_50_ > 5,000).

**FIGURE 2:**
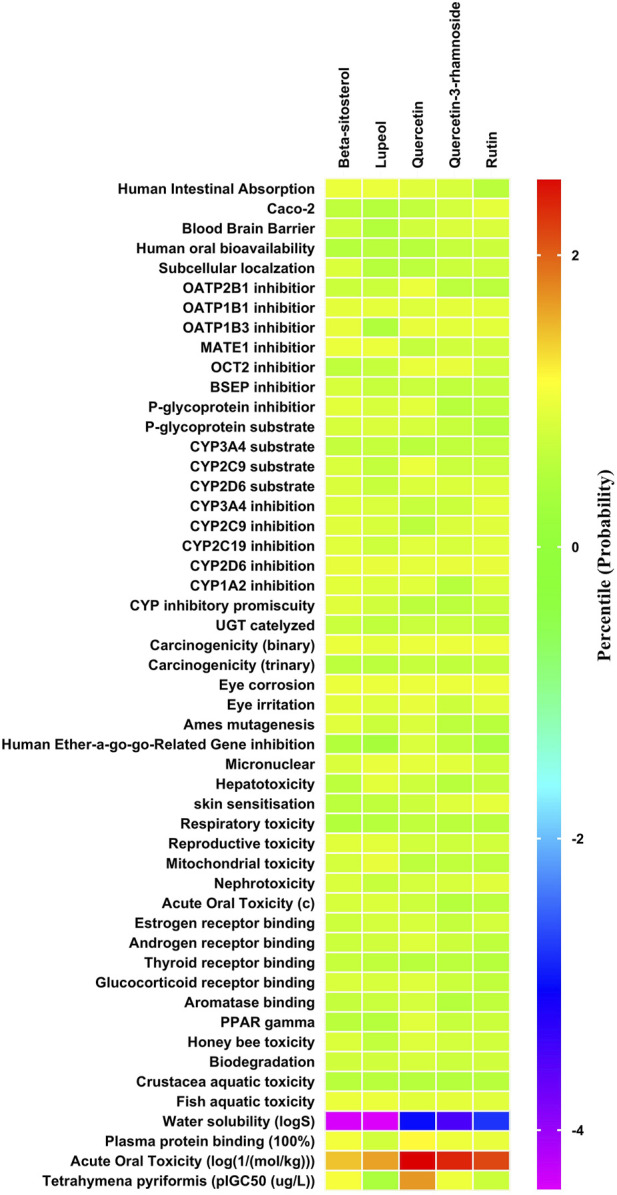
ADMET (Absorption Distribution Metabolism Excretion and Toxicity) profile of phytoconstituents (By admetSAR2.0 web tool) The Heat map depicts ADMET entities of phytoconstituents computed on the basis of probability data. Graph Pad Prism 9.0 software was utilized to create the heat map.

The ADMET patterns of particular phytoconstituents are depicted in the heatmap (Figure 2). Likewise, forecasts for druglikeness and Blood-Brain Barrier (BBB) scores were made for all five phytoconstituents, where rutin and β-sitosterol achieved the highest Drug Likeness Scores (DLS) (Table 4).

**TABLE 4:** Drug-likeness property of phytoconstituents (Data generated by MolSoft web tool).

S.No.	Phytoconstituents	Molecular formula	MW	NHBA	NHBD	MolLogP	MolLogS		BBB score	DLS
							Log (moles/L)	mg/L		
1	Lupeol	C_30_H_50_O	426.39	1	1	8.35	−6.31	0.21	3.88	−0.22
2	Quercetin	C_15_H_10_O_7_	302.04	7	5	1.19	−2.19	1952.89	2.55	0.52
3	Quercetin-3-rhamnoside	C_21_H_20_O_11_	448.10	11	7	0.32	−1.80	7,129.44	1.66	0.82
4	Rutin	C_27_H_30_O_16_	610.15	16	10	−1.55	−1.75	10775.79	1.21	0.91
5	β -sitosterol	C_29_H_50_O	414.39	1	1	8.45	−6.34	0.19	3.94	0.78

MW-Molecular weight, NHBA-Number of Hydrogen Bond Acceptor, NHBD-Number of Hydrogen Bond Donor, BBB-Blood-Brain Barrier, DLS-Drug-likeness Score.

### 3.3 Pathway and network analysis

**FIGURE 3:**
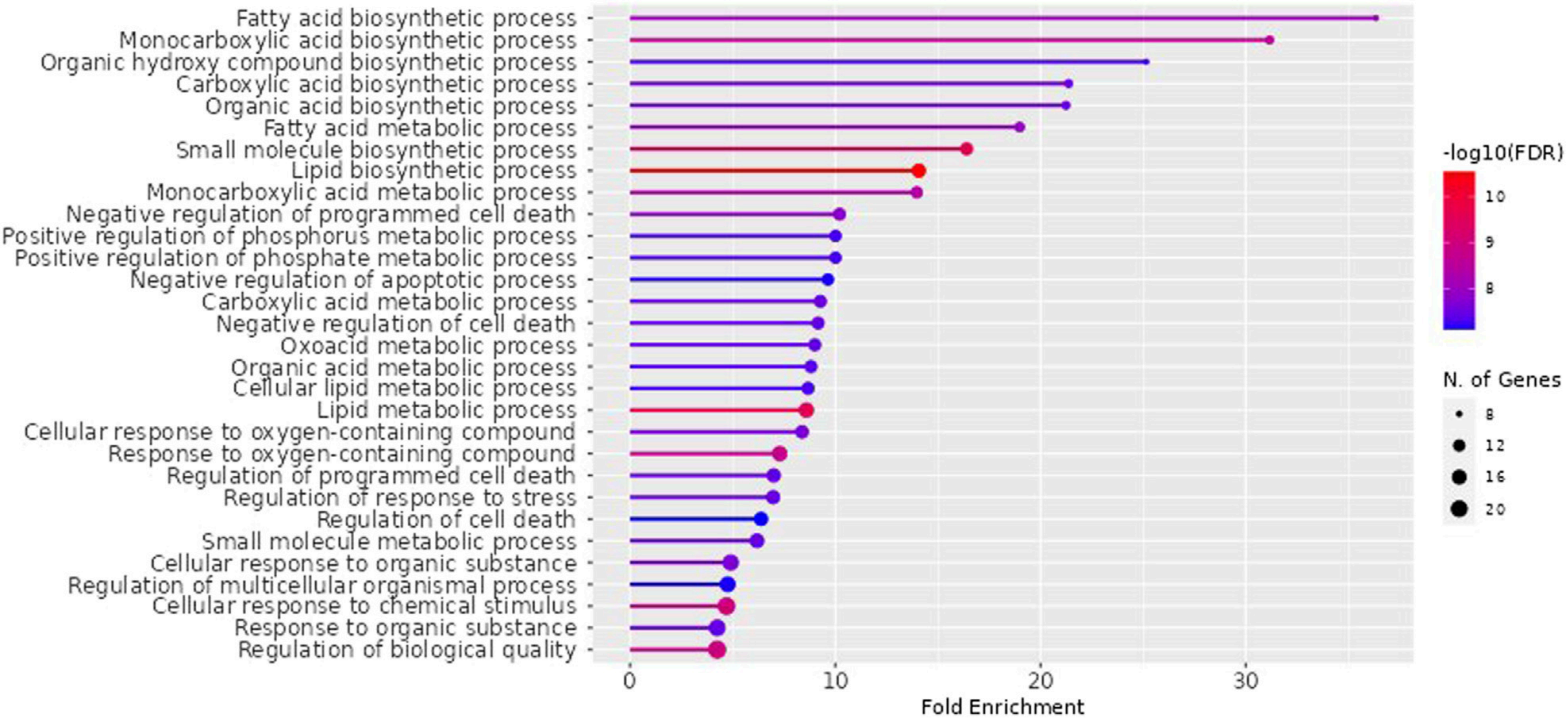
Lollipop diagram of biological processes of Gene Ontology (GO) enrichment analysis (By ShinyGO v0.741 web tool). The illustrative depiction offers valuable insights into the dynamic regulatory landscape of biological pathways. More specifically, it focuses on the intricate web of Gene Ontology (GO) enrichment analysis results, highlighting those pathways that are under the influence of proteins closely associated with NAFLD. FDR: False discovery rate.

NAFLD-related proteins were searched for in STRING. The KEGG database was analysed to uncover the NAFLD-related pathways. Gene set evaluation revealed 30 pathways that are regulated by NAFLD-associated proteins (Figure 3). Among them, “fatty acid biosynthetic process” was identified to score the highest fold enrichment with “cellular response to chemical stimulus” and “regulation of biological quality” having the highest count of gene sets (Figure 3).

**FIGURE 4:**
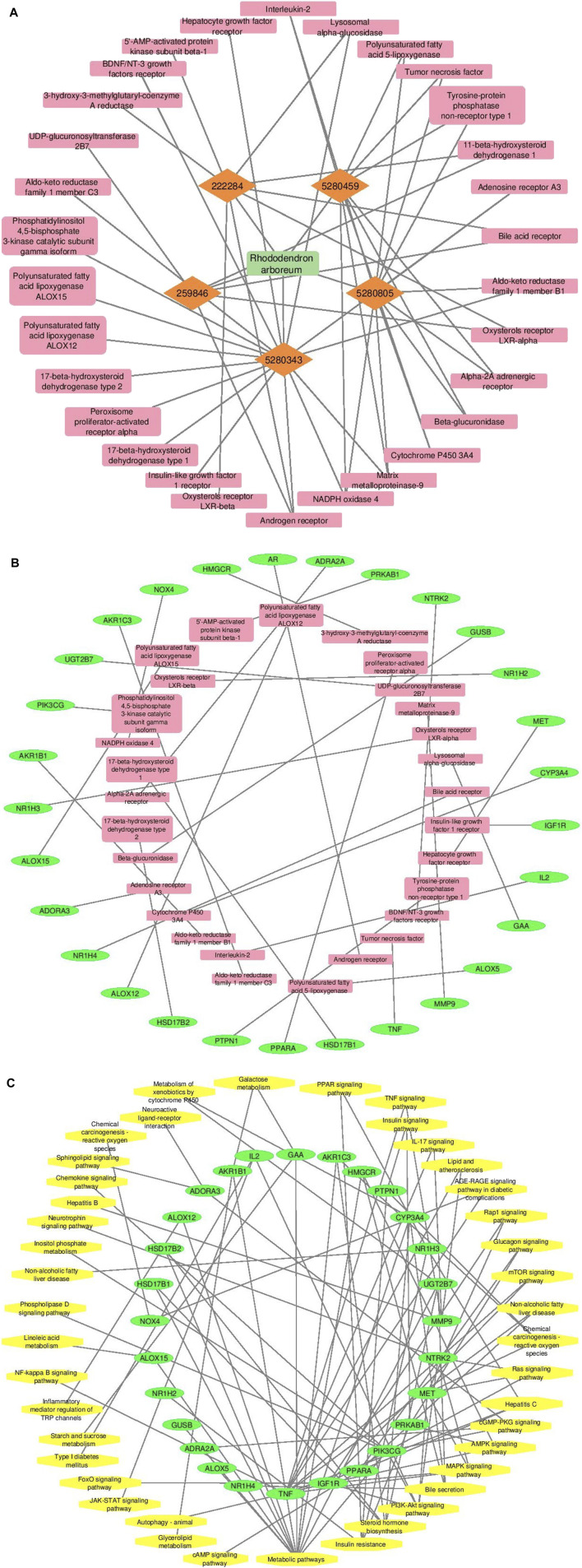
A graphical depiction of the connections and interactions among phytoconstituents, targets, genes, and pathways in a network format (By Cytoscape v3.10.0 software) **(A)** Network representation of interaction between phytoconstituents and targets **(B)** Network representation of interaction between targets and genes **(C)** Network representation of interaction between genes and pathways. Orange color represents active phytoconstituents of RAF against NAFLD, pink color represents key targets/protein of NAFLD modulated by RAF phytoconstituents, green color depicts genes of key targets/protein of NAFLD and yellow color depicts pathways mediated by genes involved in NAFLD.

**FIGURE 5:**
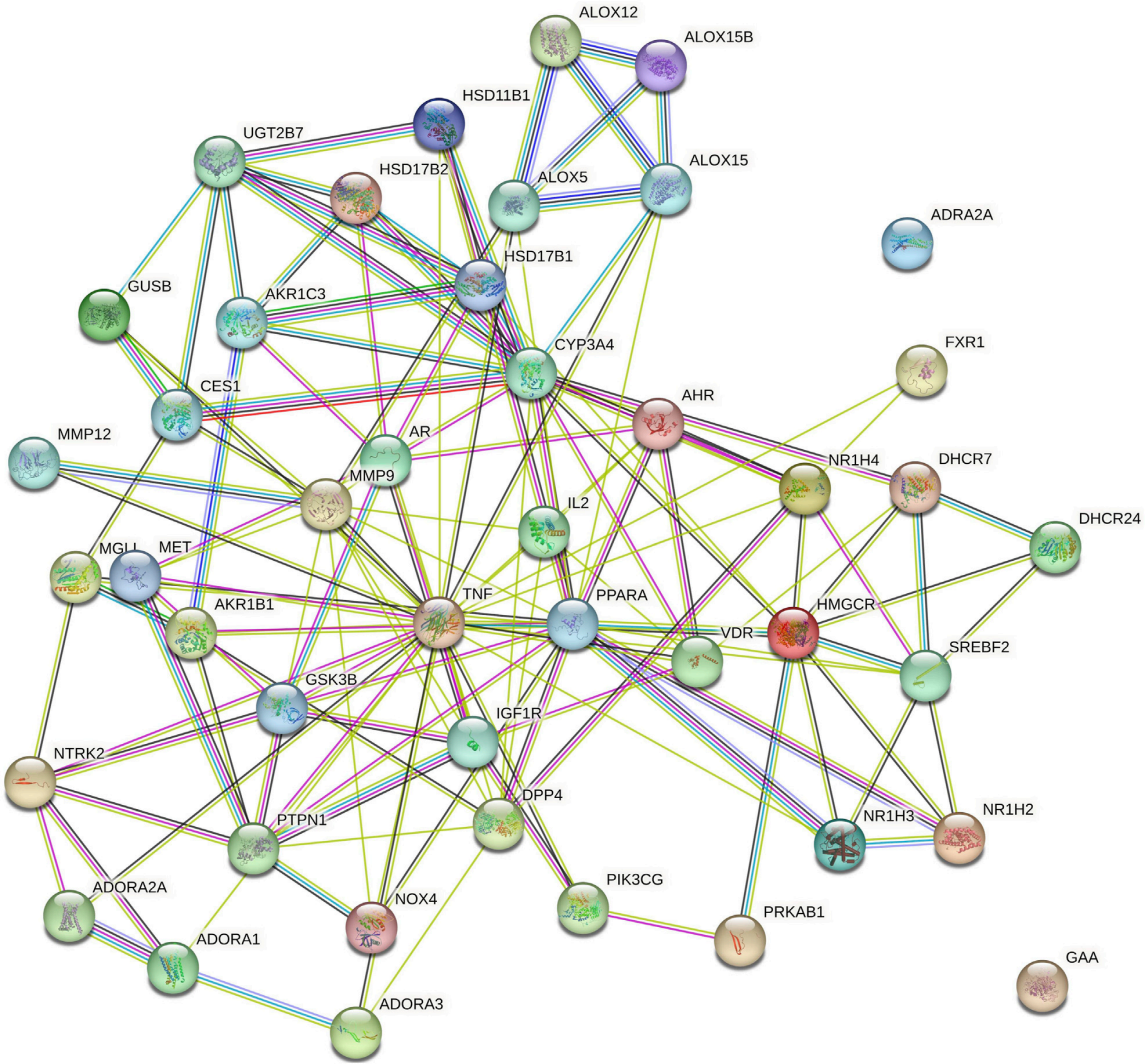
Protein-protein interaction (PPI) among genes of NAFLD targeted by RAF phytoconstituents (By STRING database webtool) The illustration portrays the interaction among proteins associated with NAFLD, as represented in the STRING database. This comprehensive depiction has been derived from data sourced from the STRING database, which is renowned for its ability to unravel complex protein-protein interactions.

**FIGURE 6:**
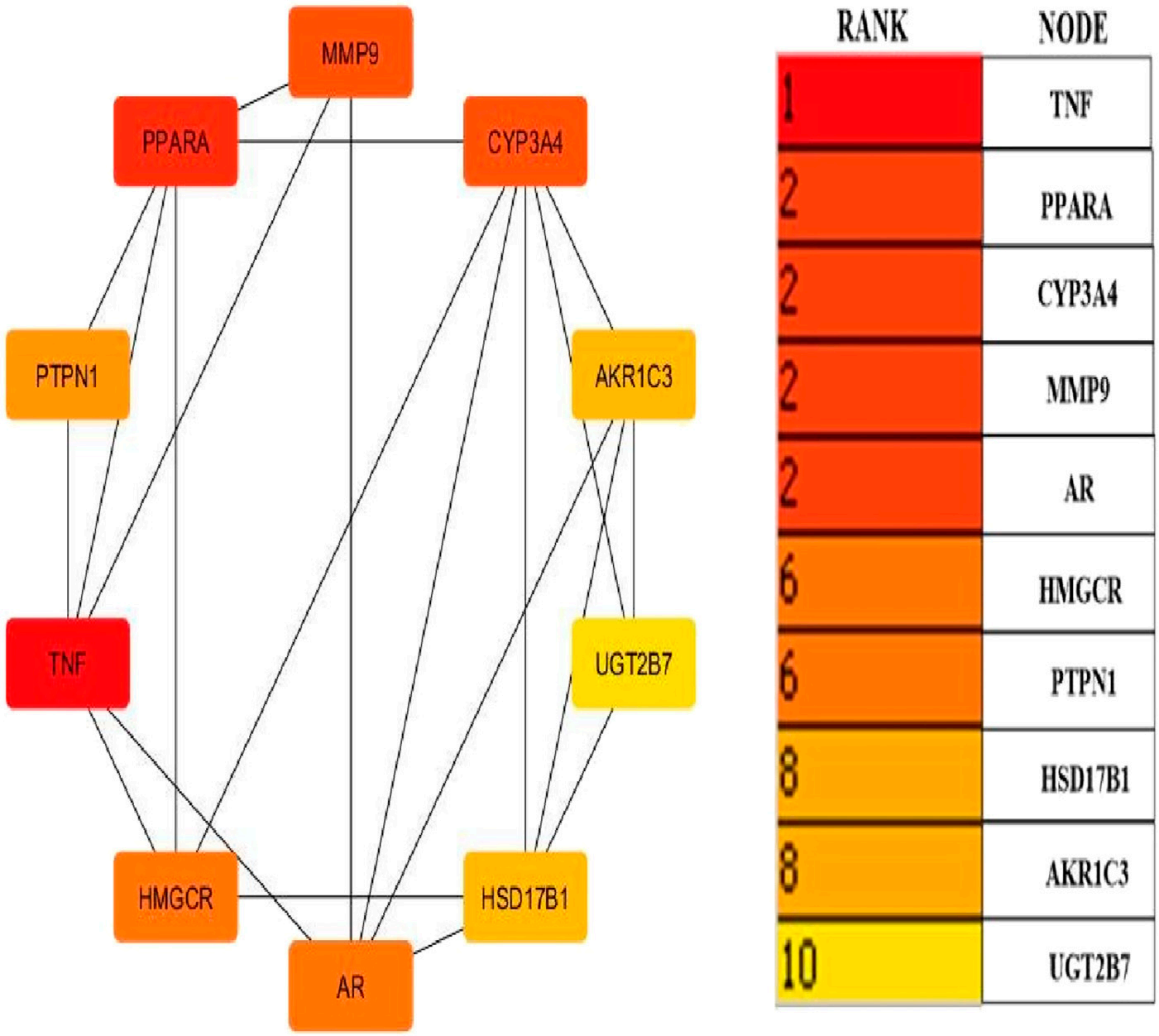
Top ten core regulatory genes of NAFLD computed by degree method (By Cytoscape v3.10.0 software) The straight lines in the illustration indicate the interaction among ten genes.

Cytoscape v3.10.0 was harnessed to intricately craft the network, interweaving RAF phytoconstituents, protein targets, genes, and the pathways that were identified (Figure 4). One hundred seven nodes spanning forty-three pathways, twenty-nine genes, thirty targets, and five phytoconstituents were included in the ultimate network. The protein-protein interaction (PPI) was represented by the STRING database (Figure 5). After constructing the PPI network, the key regulatory genes employing the CytoHubba plugin of Cytoscape v3.10.0 were assessed. The top 10 NAFLD genes (Figure 6) were selected (TNFα, PPARα (Peroxisome Proliferator-Activated Receptor Alpha), CYP3A4 (Cytochrome P450 3A4), MMP9 (Matrix Metalloproteinase-9), AR (Androgen Receptor), HMGCR (3-hydroxy-3-methyl-glutaryl-coenzyme A reductase), PTPN1 (Protein Tyrosine Phosphatase Non-Receptor Type 1), HSD17B1 (17β-Hydroxysteroid Dehydrogenase), AKR1C3 (Aldo-Keto Reductase Family 1 Member C3), and UGT2B7 (UDP Glucuronosyltransferase Family 2 Member B)) based on degree method score (Table 5).

**TABLE 5:** Top 10 genes ranked by degree method (By CytoHubba plugin software).

S.No.	Gene name	Score
1	TNF_α_	185
2	PPAR_α_	181
3	CYP3A4	169
4	MMP9	151
5	AR	138
6	HMGCR	114
7	PTPN1	107
8	HSD17B1	96
9	AKR1C3	74
10	UGT2B7	60

### 3.4 Docking studies

Through Network Pharmacology (CytoHubba plugin), the top 10 NAFLD proteins exhibiting the greatest interaction with RAF phytoconstituents were identified. In addition, based on a literature review, seven proteins with a substantial role in NAFLD and also having a positive interaction with the RAF were incorporated into the research.

**FIGURE 7:**
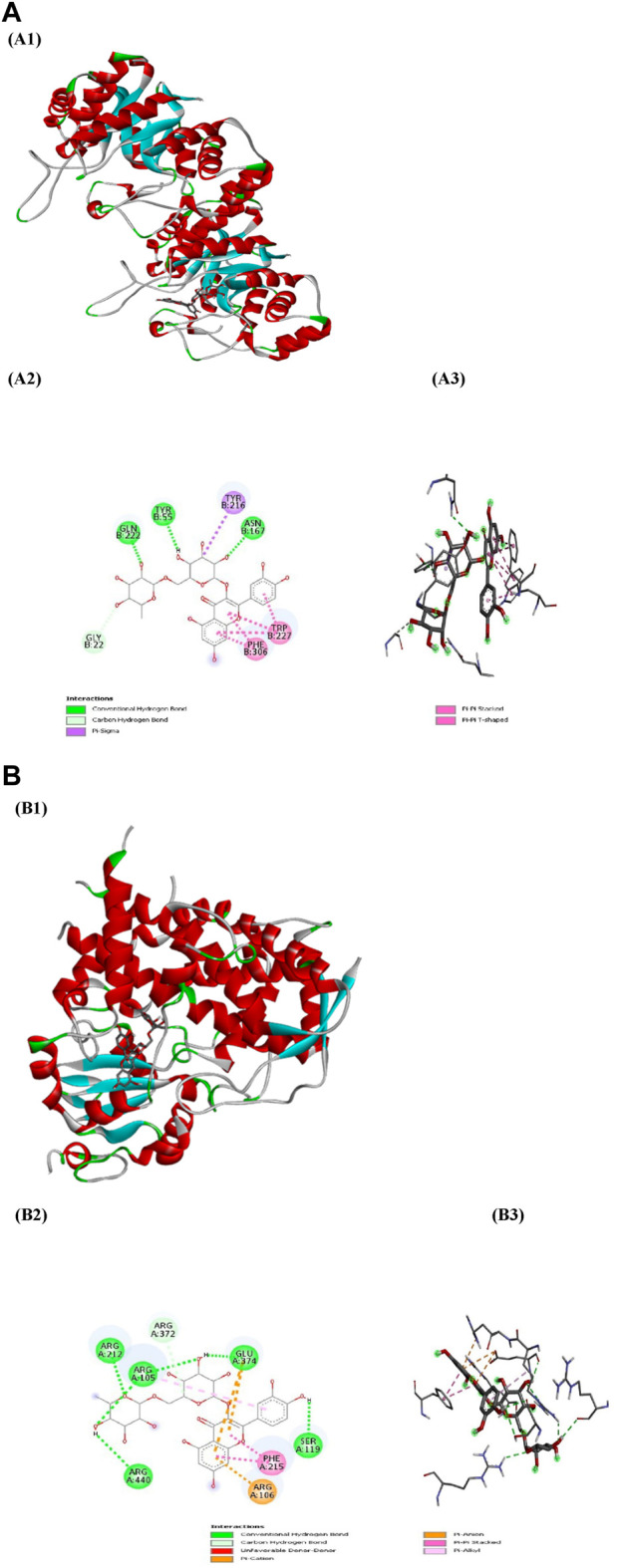
**(A)** Docking patterns of AKR1C3 target protein-Rutin **(A1)** Rutin-AKR1C3, **(A2)** 2D plot of Rutin-AKR1C3 interaction, **(A3)** 3D plot of Rutin-AKR1C3 interaction **(B)** Docking patterns of CYP3A4 target protein-Rutin **(B1)** Rutin-CYP3A4, **(B2)** 2D plot of Rutin-CYP3A4 interaction, **(B3)** 2D plot of Rutin-CYP3A4 interaction.

PyRx served as a pivotal tool for the prognostication of binding affinities between the bioactive constituents of RAF and their respective target proteins. The RAF bioactives (Lupeol, Quercetin, Quercetin-3-rhamnoside, Rutin and β -sitosterol) were docked with 17 common target proteins of NAFLD whose binding affinity results are depicted in Table 6. Binding energy is a crucial parameter in molecular docking studies as it quantifies the strength of interaction between a protein receptor and a ligand (small molecule). Molecular docking predicts the binding pose and affinity of a ligand within a protein’s binding site and estimates the binding energy associated with this interaction (Pantsar and Poso, 2018). Out of five phytoconstituent of RAF, rutin exhibited the overall lowest binding energy in all 17 target proteins of NAFLD with the lowest binding energy of −11.0 kcal/mol for AKR1C3 (RMSD: 1.934 Å) (Figure 7A) and CYP3A4 (RMSD: 2.165 Å) (Figure 7B) target proteins. Figure 7A1 illustrates the interaction between Rutin (Phytoconstituent) and AKR1C3 (Target protein). Furthermore, Figure 7A2 presents a 2D plot illustrating the Rutin- AKR1C3 interaction. The amino acid residues involved in this interaction comprise GLN (B:222), TYR (B55), TYR (B:216), ASN (B; 167), TRP (B; 227), PHE (B:306), GLY (B; 22). The intermolecular interactions identified encompass conventional hydrogen bonds (depicted in green), pi-pi stacked (shown in pink), carbon hydrogen bonds (displayed in grey), pi-pi T-shaped (presented in pink), and pi-sigma (also illustrated in pink). Figure 7B1 showcases the interaction between Rutin (Phytoconstituent) and CYP3A4 (Target protein). Additionally, Figure 7B2 provides a 2D plot illustrating the Rutin-CYP3A4 interaction. The amino acid residues participating in this interaction include ARG (A:440), ARG (A:212), ARG (A:105), ARG (372), GLU (A:374), SER (A:119), PHE (A:215), and ARG (A:106). The identified intermolecular interactions involve conventional hydrogen bonds (illustrated in green), pi-pi stacked (depicted in pink), carbon hydrogen bonds (displayed in grey), pi-anion and pi-cation (presented in yellow), and pi-alkyl interactions. The inference is based on the observation that among all the screened phytoconstituents across 17 target proteins associated with NAFLD (Table 6), rutin (the ligand) exhibited the lowest average binding affinity when compared to the others (Supplementary Material). In molecular docking studies, a low binding energy signifies a stronger interaction between a ligand and its receptor. A lower (more negative) binding energy indicates a more stable and favourable binding between the ligand and the protein receptor. This implies that the ligand has a higher affinity for the protein’s binding site (Meng et al., 2011; Pantsar and Poso, 2018).

**TABLE 6:** Binding affinity of potential active compounds in *Rhododendron arboreum* flower and their key target proteins involved in NAFLD.

Target protein	Target proteins (PDB ID)	Phytoconstituents Binding affinity (kcal/mol)				
		Lupeol	Quercetin	Quercetin-3-rhamnoside	Rutin	β -sitosterol
TNF_α_	7kp9	−9.7	−8.8	−8.6	−8.3	−8.3
PPAR_α_	6kxx	−8.3	−8.7	−7.0	−7.1	−7.1
CYP3A4	5a1r	−8.7	−8.7	−7.8	−11	−9.6
MMP9	1itv	−8.7	−7.7	−8.2	−9.4	−8.5
AR	1e3g	−8.0	−9.1	−8.4	−7.9	−7.8
HMGCR	1dq8	−8.8	−8.4	−8.7	−8.7	−7.8
F_X_R	3gd2	−7.6	−9.3	−8.9	−10.0	−9.0
HSD17B1	1a27	−9.1	−10.2	−9.8	−9.4	−8.1
AKR1C3	6gxk	−11.9	−9.4	−10.2	−11	−10.9
UGT2B7	2o6l	−9.4	−9.7	−8.4	−9.7	−7.7
PKB	1q61	−7.7	−9.0	−8.3	−7.6	−7.4
NFK-B	1nfk	−7.9	−6.8	−7.8	−7.2	−6.8
mTOR	4drh	−9.5	−7.9	−8.7	−9.1	−8.4
PI3K	1e8y	−9.6	−8.4	−9.4	−10.7	−8.8
HO-1	1s13	−8.2	−7.9	−8.2	−8.5	−7.7
PPAR_δ_	1gwx	−8.3	−8.0	−8.2	−7.4	−8.5
NRF-2	2lz1	−8.1	−6.4	−6.1	−7.9	−6.4

### 3.5 Molecular dynamics simulation

Molecular dynamics simulations were employed to gain a deeper understanding of the stability of protein-ligand complexes. In this investigation, AKR1C3 (6gxk)-Rutin and CYP3A4 (5a1r)-Rutin complexes were chosen based on docking results and subjected to a 100 ns molecular dynamics simulation. This extended analysis aimed to assess the movement, path, structural characteristics, binding affinity, and any changes in the conformation of these molecules.

**FIGURE 8:**
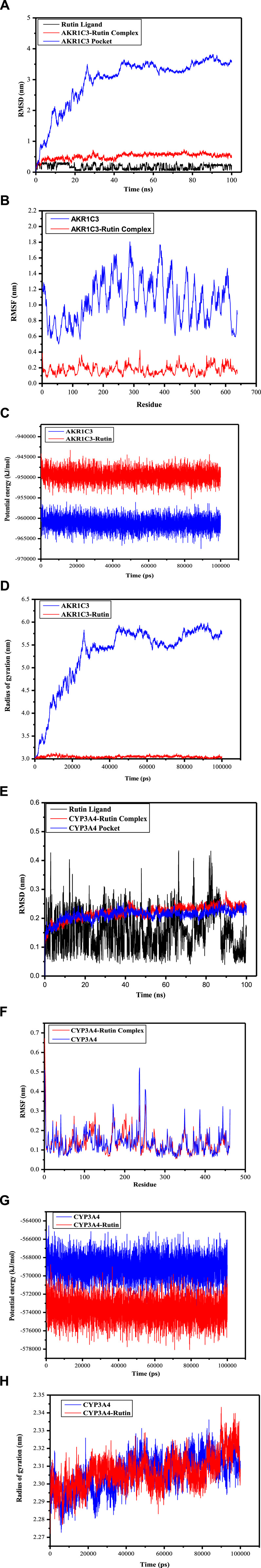
Root-mean-square deviation (RMSD), Root-mean-square fluctuation (RMSF), Potential energy (GROMACS Energies) and Radius of gyration (total and around axes) plots during molecular dynamics simulation. **(A)** The RMSD of AKR1C3 (6gxk)-Rutin Rutin Ligand displays RMSD graph for Phytoconstituent (Rutin) AKR1C3-Rutin complex displays RMSD graph for target protein (AKR1C3) with ligand (Rutin)AKR1C3 Pocket displays RMSD graph for target protein (AKR1C3) without ligand **(B)** The RMSF of AKR1C3 (6gxk)-Rutin AKR1C3 portrays RMSF graph for target protein (AKR1C3) without ligand AKR1C3-Rutin portrays RMSF graph for target protein (AKR1C3) with ligand (Rutin) **(C)** Potential energy (GROMACS Energies) of AKR1C3 (6gxk)-Rutin AKR1C3 portrays Potential energy graph for target protein (AKR1C3) without ligand AKR1C3-Rutin portrays Potential energy graph for target protein (AKR1C3) with ligand (Rutin) **(D)** Radius of gyration (total and around axes) of AKR1C3 (6gxk)-Rutin AKR1C3 portrays Radius of gyration graph for target protein (AKR1C3) without ligand AKR1C3-Rutin portrays Radius of gyration graph for target protein (AKR1C3) with ligand (Rutin) **(E)** The RMSD of CYP3A4 (5a1r)-Rutin Rutin Ligand depicts RMSD graph for Phytoconstituent (Rutin) CYP3A4-Rutin complex depicts RMSD graph for target protein (CYP3A4) with ligand (Rutin) CYP3A4 Pocket depicts RMSD graph for target protein (CYP3A4) without ligand **(F)** The RMSF of CYP3A4 (5a1r)-Rutin CYP3A4 represent RMSF graph for target protein (AKR1C3) without ligand CYP3A4-Rutin Complex represent RMSF graph for target protein (AKR1C3) with ligand (Rutin) **(G)** Potential energy (GROMACS Energies) of CYP3A4 (5a1r)-Rutin CYP3A4 portrays Potential energy graph for target protein (CYP3A4) without ligand CYP3A4 (5a1r)-Rutin portrays Potential energy graph for target protein (CYP3A4) with ligand (Rutin) **(H)** Radius of gyration (total and around axes) of CYP3A4 (5a1r)-Rutin CYP3A4 portrays Radius of gyration graph for target protein (CYP3A4) without ligand CYP3A4-Rutin portrays Radius of gyration graph for target protein (CYP3A4) with ligand (Rutin).

The Root Mean Square Deviation (RMSD) serves as a valuable metric for evaluating the stability of protein and ligand conformations, representing the degree of atom position deviation from their initial states. A reduced RMSD signifies enhanced conformational stability, and the alterations in RMSD values for these complexes were examined. As shown in Figure 8A, the RMSD of the AKR1C3 (6gxk)-Rutin complex fluctuated in the early stage and stabilized later. Although the RMSD trajectory of the CYP3A4 (5a1r)-Rutin complex fluctuated, it eventually became stable (Figure 8E). Based on the RMSD values for both the ligand and the binding pocket, it can be inferred that the active sites of small molecules and proteins maintained a state of stability. This suggests that when the small molecule ligand binds to the protein, the protein’s conformation remains relatively unchanged, indicating a stable binding interaction. Reaching the conclusion that the protein’s conformation remains relatively unchanged upon binding of the small molecule ligand, suggesting a stable binding interaction, involves various observations and analyses in molecular dynamics simulations which includes analyzing ligand-protein interactions (Figure 7), radius of gyration (Figures 8D, 8H), energy landscape and potential energy surface (Figures 8C, 8G), RMSD (Figures 8A, 8E) and RMSF (Figures 8B, 8F). Proteins that undergo only small structural changes when they bind ligands, ensuring a stable binding relationship. This provides more evidence that the binding event takes place with little to no change to the protein’s overall structure. It is indicated that the binding is accommodated within the current protein structure without major modifications when a protein either keeps its natural structure or goes through little changes following ligand binding. The fact that the protein’s shape does not change much after binding suggests that the relationship is stable and that the ligand fits well with the protein’s structure without making big changes to it. The results show that the ligands remained stable inside the cavities throughout the simulation, just as the molecular docking poses predicted. No noticeable impact of temperature or pressure on the conformation of the structure was identified.

The RMSF analysis focuses on certain regions of proteins that exhibit structural deviations from their average, a feature frequently impacted by ligand interactions. The structural changes that occur naturally result in mobility patterns that will ultimately determine ligand interaction (Martínez, 2015). The mobility patterns and changes in individual residues indicate the strength of flexibility. The higher levels of RMSF in the residues and residue groups indicate a high probability of ligand interaction because the residues are more flexible. In such a scenario, the residues are likely to change their pattern to accommodate the ligand making them more likely to resonate with the ligand molecules. The higher the RMSF, the greater the mobility or conformation changes in the residues. The increased mobility will lead to effective binding because the residues have a better chance of accommodating, and ligands may connect the residues. The lower levels of RMSF in the residues indicate less flexibility. The reduced flexibility will result in reduced mobility or adaptability. Their mobility can be minimized, leading to their inability to attach to the ligand. Figures 8B, F portray the RMSF of the AKR1C3 (6gxk)-Rutin complex and CYP3A4 (5a1r)-Rutin complex respectively. The area around the active site of the residues depicts marked variation leading to the prominent peaks in the residue. The fluctuating peaks outside the active site of the residues highlight an increased interaction potential indicating that the ligands successfully adjusted to the binding region of the target protein. The large peaks of increased fluctuations beyond the active sites of residues show that the interaction potential is high suggesting ligands versatility in their interaction with the protein’s binding pocket. The increased fluctuations outside the active site indicate the ability of residues to interact with ligands. These interactions suggest that these residues have an affinity with the ligands and accompany them into the protein structure, even though they are not directly involved in the main binding site as these interactions are beyond the active sites of the residues. By fitting properly into the larger protein binding pocket, ligands were able to connect with more residues that were outside the active site (Sharma et al., 2021). The potential energy values ranged from −955545.375 kJ/mol to −960189.625 kJ/mol for AKR1C3 and AKR1C3-Rutin, and from −945826.875 kJ/mol to −950006.0625 kJ/mol, respectively (Figure 8C). Figure 8G illustrates that the values for CYP3A4 and CYP3A4-Rutin ranged from −567295.125 kJ/mol to −568807.875 kJ/mol and −573403.1875 kJ/mol to −570852.9375 kJ/mol, respectively. The fluctuations that have been observed can be interpreted as signs of changes in the structure, stability, or interactions of the system throughout the simulation. Figure 8D depicts the Rg values for AKR1C3 and AKR1C3-Rutin, which varied between 2.96 and 5.73 nm and 2.96 and 3.05 nm, respectively. Similarly, the Rg values for CYP3A4 and CYP3A4-Rutin varied between 2.27 and 2.31 nm and 2.27 and 2.30 nm, respectively (Figure 8H). The calculated values during simulation are used to highlight protein compactness.

## 4 Discussion

NAFLD is a global health problem as it is a chronic liver disease in clinical practice (Mantovani and Dalbeni, 2021). The comorbidity of diabetes and obesity makes it a globally more worrying public health concern (Pouwels et al., 2022). The therapy for NAFLD should prioritize restoring normal hepatic activities along with reducing insulin resistance, which is a significant contributor to the disease’s development.

Network pharmacology and other bioinformatics strategies can be used as initial monitoring tools for traditional medications in managing NAFLD. Andrew L. Hopkins predicted in 2007 a paradigm shift in the pharmaceutical development field with the emergence of the “network pharmacology” concept (Hopkins, 2007; Hopkins, 2008). Considerable effort has been directed to the network pharmacology application in order to elucidate the molecular mechanisms by which traditional medications function in the treatment of complex disorders (Khanal et al., 2019).

*Rhododendron arboreum* has a role in hepatic marker enzyme pathways, inflammation, and oxidative stress (Srivastava, 2012; Verma et al., 2020). The precise molecular mechanism by which *R. arboreum* helps in NAFLD management remains obscure. As a consequence, the present study utilizes a network pharmacology approach to infer the probable molecular mechanisms by which *R. arboreum* ameliorates NAFLD. Due to the absence of standardized dosages, potential adverse effects, pharmacokinetic profiles of bioactive components, and toxicity data, additional research is required to validate the effectiveness of Ayurvedic formulations. Due to these shortcomings, Ayurveda is considered complementary and alternative medicine and not a major healthcare system globally. To increase Ayurveda’s universal acknowledgment, the integration of Ayurveda with evidence-based scientific approaches must be emphasised. Therefore, the purpose of the proposed study is to screen a target-specific standardised herbal hepatoprotective bioactive against NAFLD from an activity-guided RAF selected by network pharmacology and molecular docking studies.

Triterpenoids, flavonoids, steroids, glycosides, and phenols were the major bioactive compounds of the RAF, which competed for a decisive role in influencing NAFLD by influencing HO-1 (Heme Oxygenase-1), NRF-2 (Nuclear Factor Erythroid 2-Related Factor 2), PI3K (Phosphoinositide 3-Kinase), HSD17B1, FxR (Farnesoid X Receptor), PPARα, MMP9, TNFα, PPARδ (Peroxisome Proliferator-Activated Receptor Delta), and numerous other genes, as shown in Table 1. The finding highlights that out of twenty-five bioactives in RAF only five were found to significantly modulate NAFLD-associated genes which are highlighted in Table 1.

Employing Cytoscape v3.10.0, a network of connections among phytoconstituents, their targets, genes, and putative pathways was designed (Figure 4). The finding suggests that terpenes, steroids, phenols, and flavonoids are all viable phytoconstituents that can interact with a wide variety of protein targets critical to the pathogenesis of NAFLD. Quercetin (24 connections) and rutin (15 connections), in particular, showed promise as pharmacotherapeutic agents for NAFLD because they selectively targeted maximum protein molecules within the network which is being interpreted from Figure 4.

Within the realm of NAFLD pharmacotherapy, focusing on lipid metabolic processes, particularly fatty acid biosynthesis, and targeting insulin receptors has been identified as a prominent strategy (Eslam et al., 2020a; Eslam et al., 2020b; Mantovani and Dalbeni, 2021). The present study predicts rutin for its maximum drug-likeness score (DLS) and lupeol cannot be considered as the drug due to its negative DLS (Table 4). Further the probability of rutin to cross the blood-brain barrier is minimum (Table 4) which suggest rutin has poor bioavailability (Table 2). Evidence hints that rutin may positively influence hepatic metabolism in NAFLD by acting on lipid metabolic processes, fatty acid biosynthesis, and insulin receptors, and by modulating hepatic homeostasis through participation in hepatic marker enzymes, lipid biosynthesis, inflammation, oxidative stress, and the maintenance of the immune response.

Toxicity of the phytoconstituents was predicted (Table 3) which revealed that almost all the bioactives are safe and toxicity classes are assigned to each phytoconstituents as per GHS classification of labelling of chemicals. In general, quercetin is classified as belonging to the most toxic category (Class 3), where a lower toxicity class value indicates a greater degree of toxicity for the chemical/bioactive substance.

The impacts of the RAF on NAFLD were attributed to several biological processes according to GO (Gene Ontology) enrichment analysis. These processes included the fatty acid biosynthetic process, fatty acid metabolism, lipid metabolism, regulation of cell death, response to an organic substance, cellular response to chemical stimulus, regulation of stress response, and regulation of biological quality. Among them, “fatty acid biosynthetic process” was identified to score the highest fold enrichment with “cellular response to chemical stimulus” and “regulation of biological quality” having the highest count of gene sets (Figure 3). Higher the fold enrichment; more is the contribution of the pathway in disease pathogenesis. Therefore, the fatty acid biosynthetic process is the major contributor to the NAFLD pathogenesis. Fatty acids are notably shipped to the liver from the bloodstream through the lipolysis of triglycerides in adipose tissue. This process is under the influence of insulin, which regulates adipocyte activity. Insulin resistance, characterized by impaired post-receptor signaling in adipose tissue, plays a significant role in the development of NAFLD/NASH (Non-Alcoholic Steatohepatitis). It leads to dysregulated lipolysis, resulting in an excessive influx of fatty acids to the liver (Lomonaco et al., 2012). *De novo* lipogenesis (DNL) from glucose and fructose is the next most important source of fatty acids. A study using stable isotopes showed that the heightened hepatic lipid content observed in NAFLD patients is primarily a result of increased DNL (Donnelly et al.,. 2005).

STRING database was used to depict interaction among NAFLD target protein/genes (Figure 5) and CytoHubba plugin of Cytoscape v3.10.0 was used to analyze the core regulatory genes which further leads to the selection of the top ten genes (Table 5) of NAFLD influenced by RAF based on the degree method (Figure 6).

Finally, molecular docking was used to evaluate seventeen key target proteins (TNFα, PPARα, CYP3A4, MMP9, AR, HMGCR, FXR, HSD17B1, AKR1C3, UGT2B7, PKB (Protein Kinase B), NFK-B (Nuclear Factor Kappa B), mTOR (Mammalian Target of Rapamycin), PI3K, HO-1, PPARδ and NRF-2) and active compounds, including lupeol, quercetin, quercetin-3-rhamnoside, rutin, β -sitosterol, acquired from available literature, scientific journals and traditional medicinal books. The binding affinities reported in the docking findings ranged from −6.1 to −11.9 kcal/mol, implying that all of the targets have robust docking potential with bioactive compounds (Table 6). Binding energy, a pivotal parameter in molecular docking, quantifies the interaction strength between a protein receptor and a ligand. This energy estimation predicts the ligand’s binding pose and affinity within the protein’s site. In these studies, a lower binding energy indicates a more robust interaction. A decrease in binding energy signifies a more stable and favorable ligand-protein binding, indicating higher ligand affinity for the protein’s binding site (Meng et al., 2011; Pantsar and Poso, 2018). Rutin exhibited the overall lowest binding energy in all 17 target proteins of NAFLD with the lowest binding energy of −11.0 kcal/mol for both AKR1C3 (Figure 7A) and CYP3A4 target proteins (Figure 7B). Molecular dynamics simulations were employed to delve deeper into the interactions between AKR1C3 (6gxk)-Rutin and CYP3A4 (5a1r)-Rutin. The protein-ligand complexes displayed remarkable stability at 300 K and 1 bar, and Rutin exhibited strong binding affinity with these targets, suggesting its potential role in the therapeutic benefits of RAF for NAFLD treatment (Figure 8). The relationship between stability and binding affinity lies in their correlation within protein-ligand complexes. Stability in this context refers to how the protein-ligand complex is structurally stable as well as balanced under specified temperature and pressure. The binding affinity refers to the strength with which the protein interacts with the ligand. Therefore, the strong binding affinity of Rutin against the target proteins (AKR1C3 and CYP3A4) correlates with the remarkable stability of the protein-ligand complex under specified temperature and pressure. This can be an indicator of rutin as a possible and efficient drug candidate for NAFLD treatment. Simulations were made reliable and robust as a result of a careful approach. Many simulations of the same system were conducted to ensure multiple verifications and convergence systematically. This comprehensive approach to validation ensures that the computational outcomes align with experimental observations and further provides credibility to the obtained outcomes, enabling their reliability and reproducibility. The molecular structures computed during simulation were obtained from the PDB format, which allows replication and cross-validation against available data. This approach validates the accuracy and reliability of computational models to a certain extent. Additionally, Discovery Studio was employed to examine the target protein and ligand structures for any sort of anomaly, such as absent or incomplete residues, thereby enabling a comprehensive and accurate molecular structure ranking. The above-mentioned validation process ensures the reliability and reproducibility of the simulation’s outcomes.

Following only the foot prints of computational methods for MD simulations has several constraints, such as being a model-based computational method they may oversimplify complex biological systems neglecting certain interactions within the system which may potentially lead to inaccuracies. The MD simulation computes its values based on force fields that may not be able to take into account all molecular interactions within biological systems. Further, the scope of simulations especially in the case of larger or more intricate systems is limited by computational demands and time constraints (although 100 ns of simulation time was employed in the current research). The current simulation study has certain limitations for experimental validation due to the absence of positive controls like reference compounds such as known inhibitors, agonists, *etc.*, negative controls for the apo structure, and enzymatic assays. Addressing these limitations through further research would significantly contribute to filling existing research gaps and enhancing the comprehensiveness of future studies. Integrating computational simulations with experimental validation remains crucial for a more comprehensive and reliable understanding of molecular dynamics in biological systems.

Rutin, uncovered as a key flavonoid glycoside in RAF which interacts with a significant number of protein molecules (number of protein molecules interpreted from Cytoscape software and by molecular docking binding energy) that are implicated in the pathogenesis of NAFLD; however, its bioavailability (BBB score) is a concern that can be addressed by modifying the pharmaceutical formulation. From the perspective of network pharmacology, the present study specifies the active compounds, the likely targets, genes and pathways that govern the treatment of NAFLD, thus offering a theoretical foundation for future experimental research. Keeping in mind the constraints of network pharmacology, data mining is the only way to determine the fundamental pharmacological mechanisms for the treatment of NAFLD. Currently, network pharmacology utilises various databases for bioactive mining. Numerous information sources and experimental data may lead to discrepancies in databases, despite their curation. Nevertheless, conducting *in vitro* experiments is essential to validate these hypotheses.

## 5 Conclusion

In summary, this study represents the inaugural application of bioinformatics techniques, encompassing ADMET profiling, network pharmacology, and molecular docking, for a comprehensive investigation into the pharmacological and molecular mechanisms underlying RAF in NAFLD. The aforementioned bioinformatics and computational analyses suggested that lupeol, quercetin, quercetin-3-rhamnoside, rutin, and β-sitosterol may be the principal bioactive compounds of the RAF that elicit anti-NAFLD effects. In addition, RAF may ameliorate NAFLD by minimizing pathologic damage, inflammatory responses, and oxidative stress *via* multiple pathways, such as PI3K, HO-1 and NRF-2. The present study centred on the multi-component and multi-pathway architecture of the RAF and its mechanism of action. These results are anticipated to guide the clinical implementation of RAF and its further development for the therapeutic management of NAFLD. In addition, the fundamental limitation of ayurvedic consensus was a lack of toxicity data, side effects, and pharmacokinetic profile, all of which are meticulously examined and enumerated in this study with the aid of bioinformatics tools, which strengthens the validity of results. Nevertheless, this study has limitations, as phytochemicals and drugs that interact with multiple targets may have reduced selectivity and specificity. In the current research study, multiple target proteins are modulated by the majority of phytochemicals, resulting in either therapeutic or undesirable side effects. The presence of numerous targets contributes in part to the substantial failure rates and time and money wasted in the drug discovery process. As a result, evaluating the selectivity of compounds becomes a critical aspect of drug development and repurposing efforts. Also, additional pharmacological and clinical research is required to confirm the findings. This strategy lays a cornerstone for future research on the protective mechanisms of the RAF against NAFLD and the potential uses of network pharmacology in drug discovery.

## Figures and Tables

**Table:** 

**ADMET**	Absorption Distribution Metabolism Excretion and Toxicity
**AKR1C3**	Aldo-KetoReductase Family 1 Member C3
**AR**	Androgen Receptor
**BBB**	Blood Brain Barrier
**BindingDB**	Binding Database
**CYP3A4**	Cytochrome P450 3A4
**DLS**	Drug-Likeness Score
**FXR**	Farnesoid X Receptor
**GHS**	Globally Harmonized System of Classification and Labelling of Chemicals
**GO**	Gene Ontology
**HMGCR**	3-hydroxy-3-methyl-glutaryl-coenzyme A reductase
**HO-1**	Heme Oxygenase-1
**HSD17B1**	17β-Hydroxysteroid Dehydrogenase 1
**KEGG**	Kyoto Encyclopedia of Genes and Genomes
**LD50**	Lethal Dose
**MMP9**	Matrix Metalloproteinase-9
**MTOR**	Mammalian Target of Rapamycin
**NAFLD**	Non-Alcoholic Fatty Liver Disease
**NASH**	Non-Alcoholic Steatohepatitis
**NFK-B**	Nuclear Factor Kappa B
**NRF-2**	Nuclear Factor Erythroid 2-Related Factor 2
**Pa**	Probable activity
**Pi**	Probable inactivity
**PI3K**	Phosphoinositide 3-Kinase
**PKB**	Protein Kinase B
**PPARα**	Peroxisome Proliferator-Activated Receptor Alpha
**PPARδ**	Peroxisome Proliferator-Activated Receptor Delta
**PTPN1**	Protein Tyrosine Phosphatase Non-Receptor Type 1
**PubChem CID**	PubChem Compound Identification
**RCSB**	Research Collaboratory for Structural Bioinformatics
**SMILES**	Simplified Molecular-Input Line-Entry System
**STRING**	Search Tool for the Retrieval of Interacting Genes/Proteins
**TNFα**	Tumor Necrosis Factor Alpha
**UGT2B7**	UDP Glucuronosyltransferase Family 2 Member B7

## Data Availability

The original contributions presented in the study are included in the article/Supplementary Material, further inquiries can be directed to the corresponding author
