# Supplementary material for: From petals to healing: consolidated network pharmacology and molecular docking investigations of the mechanisms underpinning Rhododendron arboreum flower’s anti-NAFLD effects
Source: Front Pharmacol. 2024 May 28;15:1366279. doi: 10.3389/fphar.2024.1366279 (PMC11165132; doi:10.3389/fphar.2024.1366279)
Supplement: Supplementary file 2 [file Table4.pdf]

Table format: **Grouped**		Group A	Group B	Group C
		Beta-sitosterol	Lupeol	Quercetin
1	Human Intestinal Absorption	0.9972	0.9970	0.9071
2	Caco-2	0.6086	0.5183	0.6417
3	Blood Brain Barrier	0.7500	0.5000	0.7750
4	Human oral bioavailability	0.5143	0.5571	0.5429
5	Subcellular localzation	0.8699	0.5245	0.5892
6	OATP2B1 inhibitior	0.7168	0.7208	1.0000
7	OATP1B1 inhibitior	0.9413	0.9354	0.8827
8	OATP1B3 inhibitior	0.9479	0.4733	0.9480
9	MATE1 inhibitior	1.0000	1.0000	0.6600
10	OCT2 inhibitior	0.6000	0.6750	0.9750
11	BSEP inhibitior	0.8278	0.6831	0.7052
12	P-glycoprotein inhibitior	0.9166	0.8206	0.9191
13	P-glycoprotein substrate	0.8292	0.8513	0.8360
14	CYP3A4 substrate	0.6600	0.6702	0.5564
15	CYP2C9 substrate	0.8404	0.6499	1.0000
16	CYP2D6 substrate	0.8509	0.6843	0.8553
17	CYP3A4 inhibition	0.8695	0.8441	0.6951
18	CYP2C9 inhibition	0.9071	0.8200	0.5823
19	CYP2C19 inhibition	0.9025	0.7320	0.9025
20	CYP2D6 inhibition	0.9485	0.9506	0.9287
21	CYP1A2 inhibition	0.9169	0.8619	0.9106
22	CYP inhibitory promiscuity	0.9046	0.7562	0.5822
23	UGT catelyzed	0.7000	0.6000	0.7000
24	Carcinogenicity (binary)	0.9900	0.9200	1.0000
25	Carcinogenicity (trinary)	0.5962	0.5755	0.6750
26	Eye corrosion	0.9948	0.9875	0.9905
27	Eye irritation	0.9387	0.8764	0.9505
28	Ames mutagenesis	0.9000	0.7100	0.8500
29	Human Ether-a-go-go-Related Gene inhibition	0.4928	0.3607	0.8410
30	Micronuclear	0.8500	0.9700	0.9300
31	Hepatotoxicity	0.5916	0.9250	0.7375
32	skin sensitisation	0.5630	0.6096	0.7447
33	Respiratory toxicity	0.5000	0.5333	0.6222
34	Reproductive toxicity	0.9111	0.9222	0.7667
35	Mitochondrial toxicity	0.8125	0.9500	0.5875
36	Nephrotoxicity	0.8450	0.6905	0.8165
37	Acute Oral Toxicity (c)	0.8316	0.8578	0.7348
38	Estrogen receptor binding	0.7492	0.8158	0.8301
39	Androgen receptor binding	0.7283	0.7545	0.8785
40	Thyroid receptor binding	0.6846	0.6232	0.5543
41	Glucocorticoid receptor binding	0.8495	0.8218	0.8851
42	Aromatase binding	0.6607	0.7053	0.8070

Table format: **Grouped**		Group A	Group B	Group C
		Beta-sitosterol	Lupeol	Quercetin
43	PPAR gamma	0.5573	0.5072	0.9001
44	Honey bee toxicity	0.8551	0.6406	0.8728
45	Biodegradation	0.7750	0.7750	0.8250
46	Crustacea aquatic toxicity	0.5500	0.5500	0.5300
47	Fish aquatic toxicity	0.9956	0.9938	0.9124
48	Water solubility (logS)	-4.3880	-4.4140	-2.9990
49	Plasma protein binding (100%)	1.0390	0.7720	1.1640
50	Acute Oral Toxicity (log(1/(mol/kg)))	1.4550	1.6440	2.5260
51	Tetrahymena pyriformis (pIGC50 (ug/L))	1.0740	0.3850	1.6900

	Group D	Group E
	Quercetin-3-rhamnoside	Rutin
1	0.8357	0.5564
2	0.7858	0.9269
3	0.8500	0.8500
4	0.6857	0.7429
5	0.7163	0.7477
6	0.5902	0.5616
7	0.9413	0.9131
8	0.9188	0.9216
9	0.7800	0.7800
10	0.9500	0.7500
11	0.6264	0.6681
12	0.5351	0.6165
13	0.6786	0.5071
14	0.6264	0.6398
15	0.7038	0.7038
16	0.8611	0.8611
17	0.7109	0.9249
18	0.8538	0.9071
19	0.8339	0.9025
20	0.9547	0.9545
21	0.5306	0.8673
22	0.5648	0.6787
23	0.7000	0.6000
24	0.9900	1.0000
25	0.6170	0.6741
26	0.9885	0.9922
27	0.7424	0.8973
28	0.5863	0.5500
29	0.6352	0.3823
30	0.9100	0.7192
31	0.5375	0.6625
32	0.8874	0.9325
33	0.5556	0.5556
34	0.7333	0.7667
35	0.6250	0.6000
36	0.8191	0.9054
37	0.5184	0.5971
38	0.6589	0.7901
39	0.7443	0.6176
40	0.5667	0.5312
41	0.7217	0.6215
42	0.5140	0.6273

	Group D	Group E
	Quercetin-3-rhamnoside	Rutin
43	0.6941	0.7234
44	0.7943	0.7731
45	0.7250	0.7750
46	0.5050	0.5450
47	0.9457	0.8993
48	-3.4970	-2.7720
49	1.0020	0.9700
50	2.3500	2.1500
51	1.0120	0.7160
