# Supplementary material for: From petals to healing: consolidated network pharmacology and molecular docking investigations of the mechanisms underpinning Rhododendron arboreum flower’s anti-NAFLD effects
Source: Front Pharmacol. 2024 May 28;15:1366279. doi: 10.3389/fphar.2024.1366279 (PMC11165132; doi:10.3389/fphar.2024.1366279)
Supplement: Supplementary file 5 [file Image1.PDF]

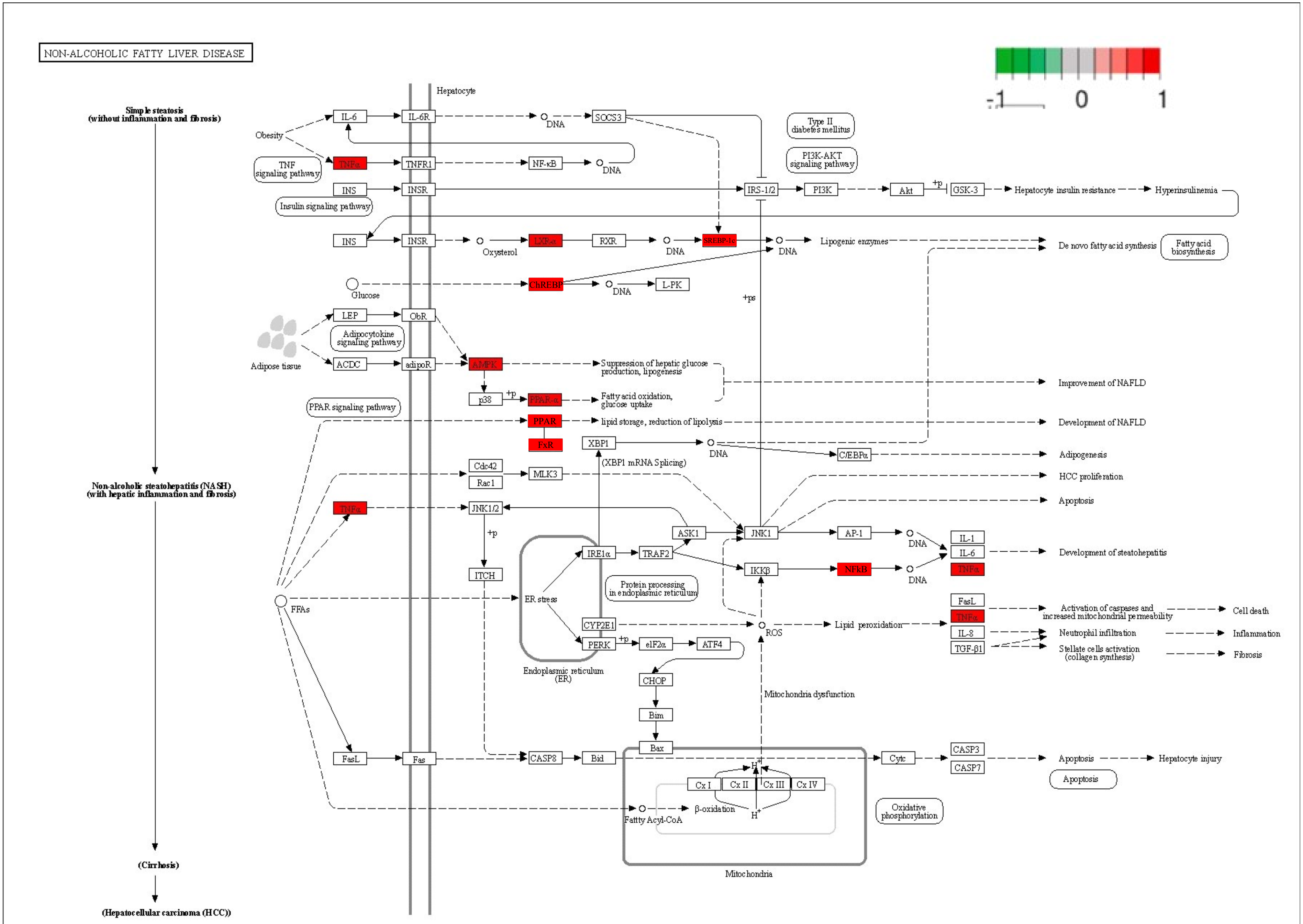

Data on KEGG graph
Rendered by Pathview
